# In Vitro Antimicrobial Potential of CAPE and Caffeamide Derivatives against Oral Microbes

**DOI:** 10.3390/ijms23084099

**Published:** 2022-04-07

**Authors:** Yin-Hwa Shih, Shih-Min Hsia, Kuo-Chou Chiu, Tong-Hong Wang, Chi-Ying Chien, Po-Jung Li, Yueh-Hsiung Kuo, Tzong-Ming Shieh

**Affiliations:** 1 Department of Healthcare Administration, Asia University, Taichung 41354, Taiwan; evashih@gm.asia.edu.tw; 2 School of Nutrition and Health Sciences, Taipei Medical University, Taipei 110301, Taiwan; bryanhsia@tmu.edu.tw; 3 Division of Oral Diagnosis and Family Dentistry, National Defense Medical Center, Taipei 11490, Taiwan; scalinghoner@yahoo.com.tw; 4 Tissue Bank, Chang Gung Memorial Hospital, Linko 33305, Taiwan; cellww@adm.cgmh.org.tw; 5 School of Dentistry, China Medical University, Taichung 40402, Taiwan; hoober0327@gmail.com (C.-Y.C.); ll820731@gmail.com (P.-J.L.); 6 Department of Chinese Pharmaceutical Sciences and Chinese Medicine Resources, China Medical University, Taichung 40402, Taiwan; 7 Department of Biotechnology, Asia University, Taichung 41354, Taiwan; 8 Chinese Medicine Research Center, College of Chinese Medicine, China Medical University, Taichung 404, Taiwan

**Keywords:** antibiotic resistance, biofilm, caffeic acid phenethyl ester (CAPE), caffeamide, minimum inhibitory concentration, minimum bactericidal concentration

## Abstract

Caffeic acid phenethyl ester (CAPE) is a natural component isolated from propolis and used in traditional medicine. We aimed to investigate the antimicrobial properties and action mechanism of CAPE and caffeamide derivatives (26G and 36M) against oral disease microbes. We resolved the minimum inhibitory and bactericidal concentrations of 26G and 36M and their stability at different temperatures and pH. We also evaluated their effect on biofilm formation and antibiotic resistance gene expression in methicillin-resistant *Staphylococcus aureus* (MRSA). Our results revealed that 26G and 36M showed the best anticancer and antimicrobial activities, respectively, compared with the other four caffeamide derivatives. Both 26G and 36M showed heat-dependent decreases in antimicrobial activity. The 36M derivative was stable irrespective of pH, whereas 26G was not stable under high pH conditions. Biofilm formation and antibiotic resistance-related gene expression were consistent with their respective phenotypes. This study provides evidence for the potential application of CAPE and caffeamide derivatives in dental medicine to cure or prevent oral diseases.

## 1. Introduction

Oral pathogenic bacteria form biofilm on the surface of teeth, leading to tissue in-flammation, gingivitis, periodontitis and tooth decay [1]. The biofilm in oral cavity is called dental plaque, which comprises many microbes attached to the mucosa, teeth, implants and dentures. It is used as an indicator of oral health and hygiene. Biofilms can protect bacteria from severe conditions with some host defense mechanisms or from applied antibiotics. *Streptococcus mutans* is a major cariogenic microbe because of its ability to attach to teeth in the initial adhesion phases of biofilm formation and create an acidic environment that corrodes the hydroxyapatite of the enamel surface. *Aggregatibacter actinomycetemcomitans* attach to teeth in the maturation phases of biofilm formation and produce the pore-forming toxin leukotoxin A, which triggers an immune response and causes periodontitis [2]. *Escherichia coli* and *S. aureus* are present in dental plaque [3,4,5]. In Amitabh Srivastava’s study, *Streptococcus spp*. was the predominant bacteria (51%) followed by *Escherichia coli* (19%) and *Veillonella* spp. (19%) in subgingival dental plaque samples [6]. *S. aureus* was detected in 18% of all participants samples from subgingival plague and the tongue [7], which is commonly present in patients with radiation caries [8]. The oral cavity has been reported as an important reservoir for *S. aureus* and MRSA [9]. Methicillin-resistant *Staphylococcus aureus* (MRSA) produces multiple antibiotic-resistant substances. The proportion of MRSA increases in the dental plaque of elderly individuals [10]. MRSA colonization was found in 22 (1.01%) of 2188 patients undergoing joint replacement surgery [11], and 77.8% of patients with oral cancer were infected with MRSA post-surgery [12]. Bacterial cells in biofilms are 1000–1500 times more resistant to antibiotics than planktonic cells [13].

Propolis is a resinous complex produced by bees, with various pharmacological properties resulting from the complexity of its composition. The components are dependent on natural or artificial vegetation; therefore, the chemical composition of propolis differs with the location. These compounds include flavonoids, phenolic acids, esters, terpenoids, steroids, and assorted amino acids [14]. Propolis displays multiple bioactivities, including antimicrobial, anti-inflammatory, anticancer, and antioxidant activities. Polyphenols and caffeic acid phenethyl ester (CAPE) are the major active compounds in propolis [15].

CAPE is found in various plants along with propolis. It exhibits anticancer properties by inhibiting angiogenesis and tumor metastasis, antioxidative, anti-inflammatory, antiviral, antibacterial, and immunomodulatory activities, reducing chemotherapy and radiation-induced damage, and improving oral wound healing effects [16]. CAPE can inhibit acid production, acid tolerance, biofilm formation, and reduce the ability of *S. mutans* to produce extracellular polysaccharides (EPS) [17]. In dentistry, propolis is used in creams, mouthwashes, and toothpaste to prevent oral diseases such as dental caries and gingivitis [18].

However, CAPE is poorly soluble in aqueous environments and is hydrolyzed to caffeic acid by esterases in vivo [19,20]. This property of CAPE limits its possible therapeutic applications, as observed by its lack of antimicrobial activity against *P. aeruginosa*, *E. coli*, *S. aureus*, and methicillin-resistant *S. aureus* (MRSA) by Arasoglu et al. [21]. To explore more stable CAPE and caffeamide derivatives, six compounds were designed, synthesized, and pharmacologically assessed in vitro as antimicrobial agents against oral microbes.

## 2. Results

### 2.1. 26G and 36M Showed the Best Potential for Antibacterial and Anticancer Activities, Respectively, than other Caffeamide Derivatives

**Figure 1:**
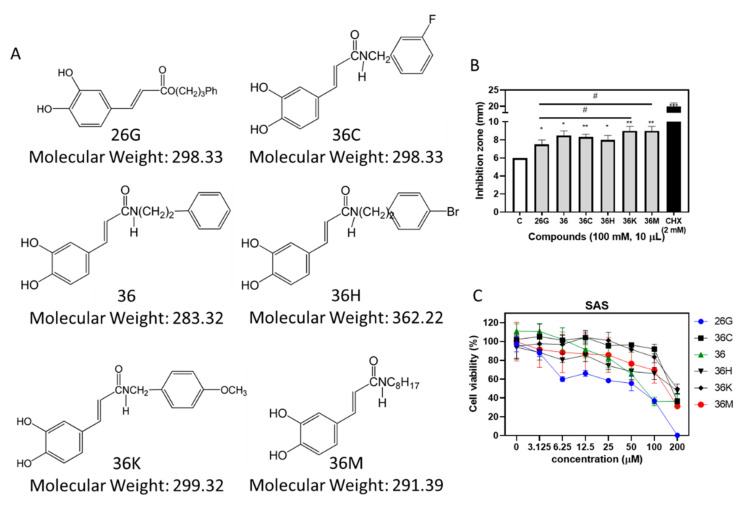
Antibacterial and anticancer activities of the caffeic acid phenethyl ester (CAPE) and caffeamide derivatives. (**A**) The chemical structures and formula of the CAPE derivatives (26G) and caffeamide derivatives (36C, 36, 36H, 36K, and 36M). (**B**) The diameter of inhibition zones of CAPE and caffeamide derivatives on *S. mutans* culture agar plates. (**C**) The cytotoxicity of caffeamide derivatives on SAS cells. Column C, dimethyl sulfoxide (DMSO); Tukey’s multiple comparisons test, compared to C, * *p* < 0.05, ** *p* < 0.01, and *** *p* < 0.001; compared to 26G, # *p* < 0.05.

We measured the inhibition zone of *S. mutans* using CAPE (26G), caffeamide derivatives (36, 36C, 36H, 36M, 36K), and cycloheximide (CHX) (Figure 1A). The data revealed significant differences in their antimicrobial properties. Ten microliter drops of the test compounds (100 mM) were added on the discs, and the average diameter of the inhibition zone from three independent experiments are shown in Figure 1B. The 36K and 36M compounds showed better disk diffusion properties than the other compounds in the test using *S. mutans*. The cytotoxicity of CAPE and caffeamide derivatives in the oral squamous cell carcinoma SAS cell line was analyzed by the 3-[4,5-dimethylthiazol-2-yl]-2,5 diphenyl tetrazolium bromide (MTT) assay. The 36M derivative showed more cytotoxic activity than 36K in SAS cells, and 26G showed the best anticancer activity (Figure 1C). Therefore, 26G and 36M were selected for further antibacterial and anticancer studies, respectively.

### 2.2. 36M Showed a Stable Antimicrobial Effect over a Wide pH Range but Weakened after Heating

**Figure 2:**
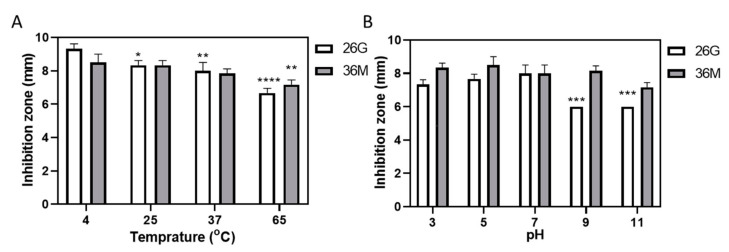
The 36M derivative was stable in terms of heat and pH. (**A**) Inhibition zones of 26G and 36M under different temperatures. The 26G and 36M compounds (100 mM, 10 μL each) were pre-incubated at 4–65 °C for 1 h before the agar diffusion test. (**B**) Inhibition zones of 26G and 36M were measured under different pH conditions. Both 26G and 36M (100 mM, 5 μL each) were pre-incubated at pH 3–11 before the agar diffusion test. Tukey’s multiple comparison test, compared to 4 °C or pH 7, * *p* < 0.05, ** *p* < 0.01, *** *p* < 0.001, and **** *p *< 0.0001.

We evaluated the stability of 26G and 36M (100 mM, 10 μL each) over a wide range of temperatures and pH. The 26G derivative showed a temperature-dependent decrease in the effect against *S. mutans* after heating. The antibacterial activity of 36M decreased upon heating to 65 °C. The antimicrobial effect of 36M was more stable than that of 26G after heating (Figure 2A). The 36M derivative also showed stability against *S. mutans* at pH 3–11; however, 26G was ineffective against *S. mutans* activity between pH 9–11 (Figure 2B). The 36M derivative exhibited better disc diffusion properties than 26G in all stability tests.

### 2.3. Determining the Minimum Inhibitory Concentration (MIC) and Minimum Bactericidal Concentration (MBC) of 26G and 36M on Oral Microbes

The MIC and MBC of 26G, 36M, and CHX were assessed using the broth dilution method for *A. actinomycetemcomitans*, *S. mutans*, *S. aureus*, and MRSA. CHX was used as positive control. The MIC and MBC of CHX were below 1 μg/mL for four tested microbes, except for the MBC of 2 μg/mL CHX for MRSA. The MIC and MBC of 26G were >400 μM for all test microbes. The MIC and MBC (shown as MIC/MBC) of 36M were 400/400, 400/400, 100/200, 200/200 μM for *A. actinomycetemcomitans*, *S. mutans*, *S. aureus*, and MRSA, respectively (Table 1). The antimicrobial activity of 36M was better than that of 26G in all test bacterial species. The MIC and MBC of 26G and 36M were consistent with that of the agar diffusion test in *S. mutans* (Figure 1B). The Gram-positive and Gram-negative were not significantly associated with the MIC and MBC of 26G and 36M. Although the growth rates of *S. aureus* and MRSA were faster than those of *A. actinomycetemcomitans* and *S. mutans*, 36M specifically inhibited the growth of *S. aureus* and MRSA.

### 2.4. Microorganism Growth Is Delayed by 26G and 36M in a Concentration-Dependent Manner

**Figure 3:**
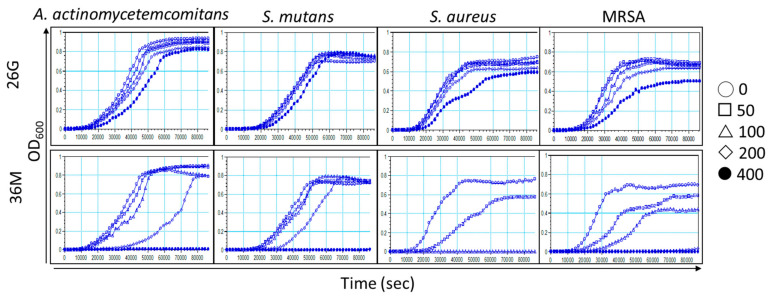
Both 26G and 36M delayed the growth of microorganisms in a concentration-dependent manner. The vehicle (DMSO) and various concentrations (50–400 μM) of 26G and 36M were used to evaluate their impact on the bacterial growth curves. *Y*-axis, OD600; *X*-axis, time (s).

The kinetic microplate method was used to analyze bacterial growth inhibition for 24 h. A log phase or stationary phase delay of the growth curve after a 24 h incubation implies that bacterial growth was inhibited or killed by 26G or 36M, respectively. The kinetic results for the 26G and 36M groups were consistent with the MIC for each microorganism (Table 1). The concentrations that affected the microorganism growth curves were consistent with the MIC (100–400 μg/mL) (Figure 3). Overall, the log phases of the microorganism growth curves were dose-dependently delayed for all the microorganism groups treated with 50–200 μM 26G and 36M. Interestingly, 26G and 36M reduced the turbidity of *S. aureus* and MRSA even in the stationary phase. This suggests that 26G and 36M specifically inhibited *S. aureus* and MRSA for at least 24 h.

### 2.5. 36M Repressed the Drug-Resistance Gene Expression of MRSA

**Figure 4:**
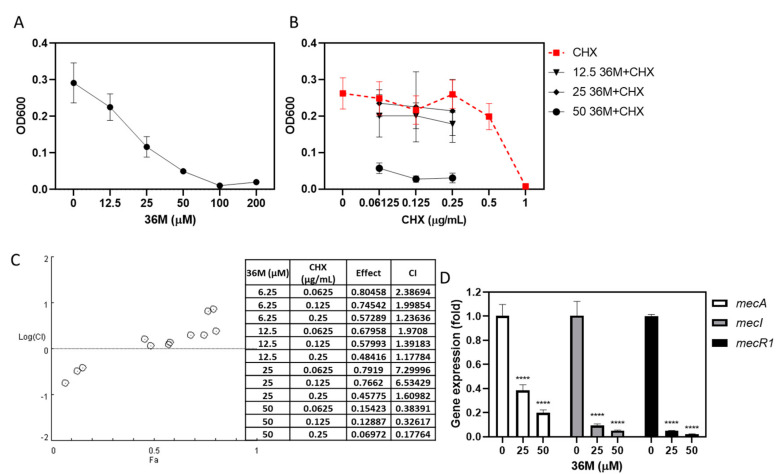
The 36M derivative reduced the resistance of MRSA to CHX. (**A**) The OD600 after MRSA was treated with various doses of 36M for 8 h. (**B**) The OD600 after MRSA was treated with various doses of CHX, and 0.06125–0.25 μg/mL CHX combined with 12.5-50 μM 36M for 8 h. (**C**) Logarithmic combination index plot of 36M and CHX co-treatment with the corresponding CompuSyn report. (**D**) Fold change in the drug-resistance-related genes *mecA*, *mecI*, and *mecR1* after treatment with 25 and 50 μM 36M in MRSA. **** *p* < 0.0001.

The MRSA were treated with various dosages of 36M (12.5–200 μM), CHX (0.06125–1 μg/mL), and 36M (12.5, 25, and 50 μM) combined with of CHX (0.06125, 0.125, 0.25 μg/mL) for 8 h. According to Figure 3, the 8 h time points were the log phase of the MRSA growth curve. At the 8 h time point, the OD600 values were 0.225 ± 0.0363, 0.117 ± 0.028, and 0.050 ± 0.004 after MRSA cells were treated with 12.5, 25, and 50 μM of 36M, respectively (Figure 4A). The OD600 were 0.249 ± 0.046, 0.217 ± 0.039, and 0.260 ± 0.039, after MRSA cells treated with 0.06125, 0.125, 0.25 μg/mL CHX, respectively (Figure 4B). The OD600 of MRSA treated with 0.06125, 0.125, 0.25 μg/mL CHX combined with 50 μM 36M significantly dropped to 0.057 ± 0.014, 0.028 ± 0.009, and 0.030 ± 0.013, respectively (Figure 4B). The synergistic and antagonistic effects of the drug combinations were analyzed and CI < 0.3 was defined as strong synergism. Accordingly, combinations of 50 μM 36M and 0.06125 μg/mL CHX, and 50 μM 36M and 0.125 μg/mL CHX showed synergistic antibacterial effects; and 50 μM 36M and 0.25 μg/mL CHX showed strong synergistic antibacterial effects (Figure 4C). Thus, treatment with 36M combined with CHX, even at low doses, significantly reduced the drug resistance of MRSA. We evaluated the expression of drug-resistance genes in 36M-treated MRSA, 36M significantly repressed the mRNA expression of *mecR1*, *mecI*, and *mecA* (Figure 4D). Although *mecI* expression was inhibited by 36M, 36M significantly inhibited *mecA* expression at 25 and 50 μM (*p* < 0.0001).

### 2.6. 36M Decreased MRSA Biofilm Formation

**Figure 5:**
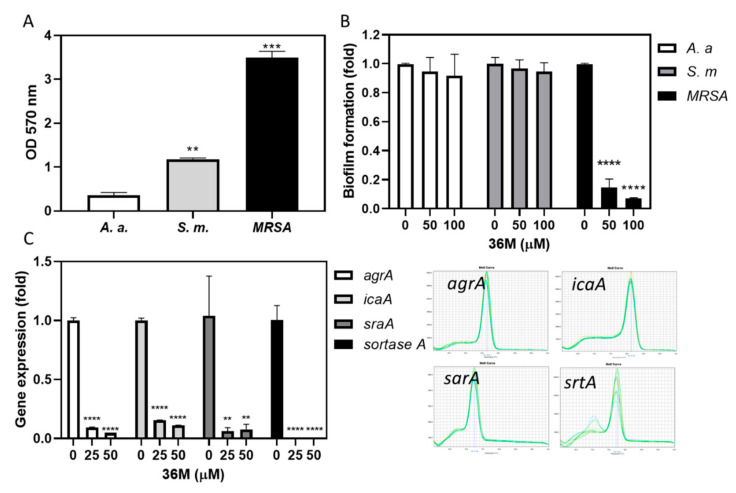
The 36M derivative reduced MRSA biofilm formation. (**A**) Biofilm-forming ability of *A. actinomycetemcomitans* (*A*. *a*.), *S. mutans* (*S*. *m*.), and MRSA. (**B**) Biofilm-formation ability of the three microbes after 36M treatments. (**C**) Left: biofilm formation-related gene expression level after 36M treatment for 48-h. Right: melting curves of *agrA*, *icaA*, *sarA*, *and srtA* amplicons. ** *p* < 0.01, *** *p* < 0.001, and **** *p* < 0.001.

The biofilm formation abilities showed that the MRSA were the strongest, the *S. mutans* were middle, and the *A. actinomycetemcomitans* were weakest after 37 °C incubation for 48 h (Figure 5A). The growth of MRSA reached the stationary phase when treated with 26G and 36M at concentrations below 200 μM for 26G, and 50 μM for 36M for 24 h (Figure 3). We used a partial inhibition dose 50 μM of 36M as the test concentration and 100 μM of 36M as the positive control concentration for 48 h in the biofilm formation assay. The 36M at 50–100 μM concentration significantly inhibited MRSA biofilm formation but did not inhibit biofilm formation of *A. actinomycetemcomitans* and *S. mutans* (Figure 5B). The RT-qPCR was used to determine the transcription levels of biofilm-related genes. After MRSA were treated with 25 μM and 50 μM of 36M, the expression of *agrA*, *icaA*, *sarA*, and srtA were reduced in *MRSA* (Figure 5C, left). The single peak in each melting curve of gene amplicons indicated that the PCR specifically amplifies the target gene fragment (Figure 5C right). The results suggested that 25–50 μM 36M suppressed biofilm formation-related gene expression of MRSA, and decreased MRSA biofilm formation ability.

### 2.7. 36M Was More Cytotoxic Than 26G and Effectively Suppressed Pro-Inflammatory Gene Expression in RAW264.7 Cells

**Figure 6:**
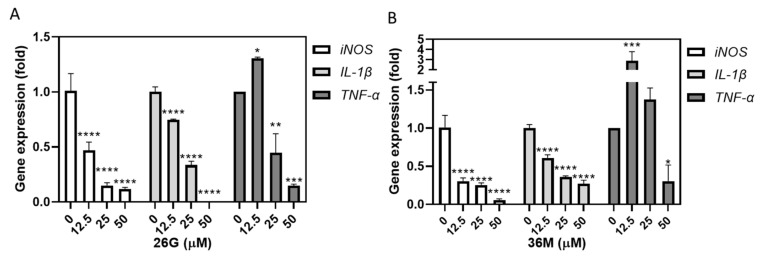
Both 26G and 36M suppressed pro-inflammatory gene expression in RAW264.7 cells. (**A**) The pro-inflammatory gene expression in RAW264.7 cells after 12.5–50 μM 26G treatment, and (**B**) 12.5–50 μM 26G treatment for 24 h. P-values were calculated by using the one-way ANOVA. * *p* < 0.05, ** *p* < 0.01, *** *p* < 0.001, and **** *p* < 0.001.

The gingival crevicular fluid is composed of a variety of immune cells, including B-lymphocytes, T-lymphocytes, monocytes, and macrophages. Macrophages are abundantly present in the inflamed gingival tissue of periodontal disease. They are thought to play an important role in eliminating microbes and releasing pro-inflammatory mediators and cytokines. Inducible nitric oxide synthase (iNOS) and interleukin (IL-1β) mRNA levels were dose-dependently inhibited in RAW264.7 cells following treatment with 36M at 12.5–50 µM and 26G at 12.5–50 µM. However, tumor necrosis factor (*TNF*)*-α* mRNA was enhanced at 12.5 µg/mL 26G and 36M treatment, but suppressed at 50 µM 26G and 36M treatment (Figure 6A,B).

## 3. Discussion

The 26G derivative, also called phenylethyl caffeate, contains an additional functional CH_2_ group compared to other caffeamide derivatives. The functional groups of the remaining caffeamide derivatives (36, 36C, 36H, 36K, 36M) contain N atoms. The experimental results showed that caffeamide derivatives tended to increase antibacterial activity and reduce cytotoxicity. However, the effect of adding F and Br atoms to the functional group was not significantly different in this study (Figure 1).

The oral cavity has a complex microenvironment in which temperature and pH change with food intake. The pH of saliva in patients with periodontal disease is significantly lower than that in normal people [22]. After sugar consumption, the pH of dental plaque is 4.5 in patients with caries [23]. In this study, we tested the antimicrobial activities of 26G and 36M against gram-positive *S. mutans* under normal and extreme temperature and pH conditions. The diameters of the inhibition zones showed that both 26G and 36M were sensitive to heat, and the antimicrobial activities were reduced. The antimicrobial abilities of 26G and 36M were stable under general physiological and low-pH conditions. The 36M derivative, unlike 26G, was stable at high pH (Figure 2A,B). The resonance of -O- in 26G increases the electronic density of the aromatic groups. Therefore, aromatic groups are easily oxidized to destroy 26G at a high pH.

CAPE can inhibit the cariogenic bacterium *S. mutans* [17]. The hexane and chloroform isolated fractions of propolis ethanolic extracts, unlike the ethyl acetate and ethanol isolated fraction, inhibited *S. mutans* [24]. This implies that CAPE might be present in the hexane- or chloroform-isolated fractions. The antibacterial activity of 36M was more effective against *S. aureus* and MRSA than against *A. actinomycetemcomitans* and *S. mutans* (Table 1 and Figure 3). The antibacterial ability of caffeamide derivatives may be different from that of CAPE in this study. The molecular weight of 26G (298.33 g/mol) and 36M (291.39 g/mol) were similar. However, 26G is more hydrophilic than 36M as the polarity of the aromatic group in 26G is stronger than that of the alkyl group in 36M. The solubility and diffusion rate of 26G were poorer than that of 36M. This could explain why the antibacterial activity of 36M was higher than that of 26G. In addition, the cell wall is composed of alkanes; therefore, the cell wall penetration ratio of 36M of the alkyl group is better than 26G, causing potent antibacterial activity.

Dental plaque is rapidly colonized by multidrug-resistant bacteria. Using 2% CHX reduced the incidence of *S. aureus* colonization and MRSA in the oral mucosa [25]. The ethanolic extract of propolis displays synergism with certain antibiotics [26]. The combinations of 0.06125–0.25 μg/mL of CHX and 50 μM of 36M significantly reduced the OD600 in the synergistic drug test. This may be because 36M inhibited *mecA* expression at concentrations above 25 μM. The synergistic effect of CHX and 36M was distinct in the log phase (Figure 4B). The mechanisms of methicillin resistance in MRSA are complex and remain unclear. For example, the drug resistance of MRSA involves the catalytic function of β-lactamase, which hydrolytically degrades these antibiotics. Methicillin resistance in MRSA is affected by *mec* and *fem* gene regulation. When MRSA comes in contact with lactams, *mecA* is activated and translated into penicillin-binding protein 2a (PBP2a). The expression of *mecA* is regulated by the upstream *mecR*, which regulates PBP2a expression and mediates the influence of methicillin resistance. *mecR* includes two genes, *mecI* and *mecR1.* The *mecI* gene is encoding a transcriptional regulator to suppress *mecA*, and the *mecR1* gene is encoding a membrane-bound signal transduction protein that activates *mecA*. The expression of drug-resistant *mec* genes was affected by 36M; 36M repressed *mecA*, *mecI*, and *mecR1* expression in a dose-dependent manner (Figure 4D). These results suggested that 36M inhibited antibiotic resistance mechanisms involving *mecA*. Despite this, decreased *mecI* expression did not increase *mecA* expression after the 36M treatment. Moreover, unknown regulators are involved in the transcriptional control of *mecA* [27]. Point mutation or deletion in full-length *mecR1* and *mecI* were detected in potent MRSA strains [28,29]. Determination of whether the promoters, genes and operons of *mecA*, *mecR1* and *mecI* are wild-type, and identification of other regulators of *mecA* in MRSA ATCC 43300, must be investigated in further studies.

The in vitro inhibitory activity of propolis extracts against Gram-positive bacteria is more effective than that against Gram-negative bacteria [30]. However, caffeamide derivative 36M demonstrated significant anti-biofilm formation activity in Gram-positive MRSA but was ineffective on Gram-negative *A. actinomycetemcomitans* and Gram-positive *S. mutans* (Figure 5B). In Gram-positive bacteria, sortase enzymes anchor the bacterial cell wall surface proteins involved in host cell attachment and biofilm formation [31]. Sortase A (srtA) is an enzyme that participated in cell wall generation in Gram-positive species, and is essential for antibiotic resistance and bacterial colonization. The expression of polysaccharide intracellular adhesin (PIA) is regulated by genes of the *staphylococcal accessory regulator* (*sarA*) and the *intercellular adhesive* (*ica*) operon in MRSA [32]. In *S. aureus*, the *accessory gene regulator* (*agr*) locus regulates most extracellular and surface-attached virulence factors. As cell density increases, *agr* becomes active, and extracellular degradative exoenzymes and toxins are produced [33]. Recent evidence reveals that repressing *srtA* decreases biofilm formation and antibiotic resistance [34,35]. We only analyzed the expression of antibiotic resistance- and biofilm formation-related genes in MRSA that caused its reduced growth (Figure 3) and biofilm formation (Figure 5B). Further investigation is required to determine whether the bactericidal mechanism of 36M differs between Gram-positive and Gram-negative microbes.

Besides the overaccumulation of dental plaque, intractable periodontal disease is usually associated with hyperimmunization, and clinically, it requires combined treatment with anti-cytokine agents [36]. Both 26G and 36M at concentrations below the MIC affected oral squamous cell carcinoma (OSCC) cell viability (Figure 1C). However, the transcription level of pro-inflammatory genes was reduced in RAW264.7 cells (Figure 6A,B). The mRNA levels of the osteoclast-related factors, such as *TNF-α*, in RAW264.7 cells were considerably induced by 12.5 µM of 26G and 36M, but reduced by 50 μM of 26G and 36M treatments in our study. This indicates that 26G and 36M doses over 50 µM suppressed macrophage differentiation into osteoclasts. Further studies are required to determine whether low doses of both CAPE and caffeamide derivatives can induce osteoclast differentiation. Overall, 26G and 36M have antimicrobial, anti-inflammatory, and osteoclast differentiation-suppressing potential, and 36M has antibiofilm-forming effects and synergistic effects with antibiotics. Both compounds might be efficient remedies for intractable oral disease prevention and therapy.

## 4. Materials and Methods

### 4.1. Antimicrobial Agents and Other Chemicals

Caffeic acid phenethyl ester (CAPE) and caffeamide derivatives (26G, 36, 36C, 36H, 36M, and 36K) were synthesized and provided by Professor Yueh-Hsiung Kuo using a previously published method [37]. Briefly, enzotriazol-1-yloxytris(dimethylamino)phosphonium hexafluorophosphate (BOP, 1.2 Equation) was dissolved in dichloromethane (5 mL) and added to a mixture of caffeic acid (0.56 mmol), phenethyl alcohol, and corresponding amines (1.2 eq) and triethylamine (80 μL) in dimethylformamide (1.0 mL). The mixture was stirred at 0 °C for 0.5 h and then stirred at room temperature for 2 h. The reaction mixture was evaporated under vacuum and partitioned between ethyl acetate (EtOAc) and H_2_O. The EtOAc layer was washed with 3 N HCl and 10% NaHCO_3_, and concentrated under vacuum. The residue was further purified by column chromatography on silica gel (70–230 and 230–400 mesh, Merck, Burlington, MA, USA). The final products (80–83% yield) were recrystallized to produce pure crystals. Their spectroscopic data were identical to the published data [37,38,39,40]. The purity of all the products was approximately >95%. Commercial 100 mg/mL chlorhexidine (CHX) was purchased from Sigma–Aldrich (Sigma-Aldrich®, St. Louis, MO, USA). All caffeamide derivatives were dissolved in dimethyl sulfoxide (DMSO) as stock solutions of 100 mM and were stored at −20 °C. The chemical structures of CAPE and caffeamide derivatives are shown in Figure 1A.

### 4.2. Microbial Cultures

*Aggregatibacter actinomycetemcomitans* (ATCC number: 33384), MRSA (ATCC number: 43300), *S. aureus* (ATCC number: 25923), and *S. mutans* (ATCC number: 25175) were used in this study. *A.* *actinomycetemcomitans* was cultured in brain heart infusion (BHI) broth, and other bacterial species were cultured in tryptic soy broth (TSB). The bacteria were inoculated by loop transfer from frozen tubes into 3 mL nutrient broth slant and were maintained at 37 °C for 24 h with constant shaking at 200 rpm. Bacteria from these cultures were transferred to agar plates and incubated overnight. Random selected single colony was transferred to an appropriate broth and incubated for 4–6 h to achieve log-phase growth. The optical density of each culture at 600 nm (OD600) was adjusted to 1.0, using fresh broth to obtain a standard inoculum of 10^8^ CFU/mL. Stock cultures were maintained at −80 °C in growth broth containing 25% sterile glycerol [41].

### 4.3. Agar Diffusion Test

Drops of 10 μL DMSO or test compounds (100 mM) were dispensed on the filter paper discs with 6 mm diameter, and the discs were air-dried overnight. After autoclave sterilization, molten 1.5% agar broth was equilibrated in a 50 °C water bath for 30 min. After spreading 100 µL 10^6^ CFU/mL microbes on the solidified plates, the dried filters were placed on the agar surface and the cultures were incubated for 24 h at 37 °C. The diameter of inhibition zone was recorded, and images were acquired [42].

### 4.4. Cell Culture

The murine macrophage RAW264.7 cell line and the oral squamous cell carcinoma (OSCC) cell line SAS were cultured as previously described [43,44].

### 4.5. Cell Viability Assay

SAS cells (10^4^ cells/100 µL) were inoculated and cultured for 20–24 h at 37 °C in 96-well tissue culture plates. For cytotoxicity analysis, the cells were treated with two-fold serial dilutions of the CAPE and caffeamide derivatives (26G, 36, 36C, 36H, 36M, and 36K) with media (3.125–200 µM) for 24 h. After removing the culture medium, the MTT assay was performed according to a previously reported protocol [45].

### 4.6. Heat Stability Test

To evaluate the stability of the 26G and 36M at different temperatures, 10 μL test compounds (100 mM) were pre-incubated at 4 °C, 25 °C, 37 °C, and 65 °C for 1 h for a heat stability test, followed by an agar diffusion test. The inhibition zone diameter was recorded [46].

### 4.7. pH Value Stability Test

The pH of the water was adjusted to 3, 5, 7, 9, and 11 using HCl or NaOH in separate tubes and measured with a pH meter before use. Then, 5 µL of test compound (100 mM) was mixed with 5 µL of water with different pH values (pH 3–11). Then, an agar diffusion test was performed [46].

### 4.8. Determination of Minimum Inhibitory Concentration (MIC) and Minimum Bactericidal Concentration (MBC)

Two milliliters of broth with various concentrations of the test compounds were prepared in 15-mL culture tubes and inoculated with 2 μL of 10^6^ CFU/mL microbes. The cells were incubated at 37 °C for 24 h with constant shaking at 200 rpm. Cycloheximide (CHX) was used as the positive control. The lowest test concentrations at which turbidity was not visible were considered to represent the MIC, then inoculated onto sterile 10-cm test compound free nutrient agar plates and incubated for an additional 24 h. The lowest concentration of the test compound at which no colony growth was considered MBC [46].

### 4.9. Growth Curve Assay

Bacterial suspensions were prepared by inoculating 1 μL of 10^6^ CFU/mL microbes from each logarithmic phase stock in 200 μL of the liquid medium containing various concentrations of 26G and 36M in 15 mL culture tubes, and 200 μL of sterile liquid broth was used as a blank. The 24 h growth curve analyses were performed for *A. actinomycetemcomitans*, *S. mutans*, *S. aureus* and MRSA at 37 °C. The kinetic analysis included a 5 s shaking step before each of the OD600 time point measurements, which were recorded at 30 min intervals, The concentration was analyzed using a VersaMax™ ELISA microplate reader (Molecular Device, San Jose, CA, USA) and Softmax^®^ Pro (version 5.4.1) software [46].

### 4.10. Drug Synergistic Test

Log phase MRSA suspensions were prepared in 2 mL of TSB in 15 mL culture tubes by inoculation with 2 μL of 10^6^ CFU/mL from MRSA standard inoculum, and incubated at 37 °C for 4–6 h with constant shaking at 200 rpm. These MRSA suspensions were treated/co-treated with 36M (12.5–200 μM), CHX (0.06125–1 μg/mL), and a combination of 36M (12.5–50 μM) and CHX (0.06125–0.25 μg/mL). The 24 h kinetic growth curve was performed for the drug synergistic test. The drug synergistic effect was calculated as 8 h turbidity (bacterial growth as log phase) [45].

### 4.11. Biofilm Formation Assay

Bacteria (10^6^ CFU/mL) were inoculated in each well of a 96-well plate. The DMSO or 50–100 µM 36M were added, mixed well, and cultured at 37 °C for 48 h. The medium was removed, and the wells were washed twice with 200 μL phosphate-buffered saline (PBS) and air-dried for 1 h. Crystal violet (150 μL of 0.1% *w*/*v*) staining was used for 10–15 min to each well. After the staining, the crystal violet was aspirated, and each well was rinsed three to four times with water. After aspiration and air-drying, 150 μL of 33% acetic acid was added to each well. Absorbance was determined at 550 nm on a VersaMax™ ELISA microplate reader [47].

### 4.12. Reverse Transcription-Quantitative Polymerase Chain Reaction

Bacteria (10^6^ CFU/mL) were inoculated at various concentrations of 36M for 24 h. Microbes were harvested for reverse transcription-quantitative polymerase chain reaction (RT-qPCR) analysis. Total RNA from the 36M-treated cells was extracted using TRI reagent (Molecular Research Center, Inc., Cincinnati, OH, USA). RT of total RNA was performed using a random primer to produce cDNA. SYBR Green Master Mix was mixed with the cDNA to perform RT-qPCR. The expression of antibiotic resistance-related genes (*mecA*, *mecI*, and *mecR1*) and biofilm formation-related genes (*srtA*, *agrA*, *icaA*, and *sarA*) was normalized to glyceraldehyde 3-phosphate dehydrogenase (*GAPDH)* expression. The primer sequence information and PCR program settings were based on previously published methods [48,49].

RAW264.7 cells were grown to 70% confluence in 60 mm dishes and were treated with 12.5, 25, and 50 µM 26G and 36M alone for 24 h. Total RNA was extracted using TRI reagent, and RT was performed using an oligo-T primer to produce cDNA. SYBR Green Master Mix was mixed with the cDNA to perform RT-qPCR. The expression of pro-inflammatory genes *iNOS*, *IL-1β*, and *TNF-α* was normalized with *GAPDH* expression. The primer sequence information and PCR program settings were using previously published methods [46,50].

### 4.13. Statistical Analysis

Data from independent experiments with three replicates in each group were analyzed using the paired *t*-test and one-way analysis of variance (ANOVA) in Prism 5.0 (GraphPad Software, Inc., La Jolla, CA, USA). One-way ANOVA was followed by Tukey’s multiple comparisons test to compare replicate mean values between all compounds in each condition. Differences between variants were considered significant at *p* < 0.05. CompuSyn software was used to analyze the synergistic and antagonistic effects of the drug combinations. CI < 0.3 is defined as strong synergism in the CompuSyn software.

## 5. Conclusions

Among the six CAPE and caffeamide derivatives, 26G showed stronger cytotoxicity but weaker antibacterial activity. The 36M derivative has weaker cytotoxicity, but better stability and antibacterial effects. Treatment with 36M effectively reduced the formation of MRSA biofilms. When 26G and 36M were combined with CHX, lower doses of CHX can be used to effectively reduce the side effects resulting from long-term use. In addition, 26G and 36M can reduce the expression of genes related to pro-inflammatory responses. Therefore, CAPE and caffeamide derivatives have potential applications in dentistry; however, appropriate CAPE and caffeamide derivatives need to be selected in accordance with the clinical applications.

## Figures and Tables

**Table 1:** MIC and MBC of 26G and 36M for the oral microbes.

	MIC/MBC		
	26G (μM)	36M (μM)	CHX (μg/mL)
*A. actinomycetemcomitans* (G−)	>400/>400	400/400	<1/<1
*S. mutans* (G+)	>400/>400	400/400	<1/<1
*S. aureus* (G+)	>400/400	100/200	1</<1
MRSA (G+)	>400/>400	200/200	1</2

G, gram-negative; G+, gram-positive; MIC, minimum inhibitory concentration; MBC, minimum bactericidal concentration; CHX, cycloheximide.

## Data Availability

Not applicable.
